# Supplementary material for: Lumican/Lumikine Promotes Healing of Corneal Epithelium Debridement by Upregulation of EGFR Ligand Expression via Noncanonical Smad-Independent TGFβ/TBRs Signaling
Source: Cells. 2024 Sep 24;13(19):1599. doi: 10.3390/cells13191599 (PMC11475839; doi:10.3390/cells13191599)
Supplement: Supplementary file 1 [file cells-13-01599-s001.zip › cells-3041284-supplementary tables.pdf]

Supplemental Table S1: Primers used in qRT-PCR of EGFR ligands

Human Gene	Forward	Reverse
HBEGF	TGGAGAATGCAAATATGTGAAGGA	AGGATGGTTGTGTGGTCATAGGTAT
TGFα	GATTCCCACACTCAGTTCTGCTT	CACAGCGTGCACCAACGT
EREG	TCCATCTTCTACAGGCAGTCC	CACGGTCAAAGCCACATACTC
BTC	AGCCTGGGAAGTAGTTTCGT	CACCACACAGTGAAGGATCA
GAPDH	CCGAGCCACATCGCTCAGACA	CTGCAAATGAGCCCCAGCCTTCTCC
AREG (VHPS-493)	GTGTGGGGAAAAGTCCATGA	CTGGAAAGAGGACCGACTCA
b-Actin	CATCTCTTGCTCGAAGTCCA	ATCATGTTTGAGACCTTCAACA

Abbreviation: EGF (Epithelium Growth Factor), HBEGFE (Heparin binding EGF), TGFα; EREG (Epiregulin), BTC (Betacellulin), and AREG (Amphiregulin)

Supplementary Table S2. Fold of upregulated expression of EGFR Ligands

2hours avg Delta CT values				
		Control	$LumC13_{C-A}$	Fold (LumC13C-A/Control)
	TGFa	7.080×10^{-3}	10.659×10^{-3}	1.5 fold
	HBEGF	3.207×10^{-3}	4.463×10^{-3}	1.4 fold
	EREG	9.338×10^{-3}	17.742×10^{-3}	1.9 fold
	EGF	1.898×10^{-5}	3.364×10^{-5}	1.8 fold
	BTC	7.588×10^{-6}	38.628×10^{-6}	5.1 fold
	AREG	5.233×10^{-2}	11.880×10^{-2}	2.3 fold
