# Supplementary figures and images for: Lumican/Lumikine Promotes Healing of Corneal Epithelium Debridement by Upregulation of EGFR Ligand Expression via Noncanonical Smad-Independent TGFβ/TBRs Signaling
Source: Cells. 2024 Sep 24;13(19):1599. doi: 10.3390/cells13191599 (PMC11475839; doi:10.3390/cells13191599)

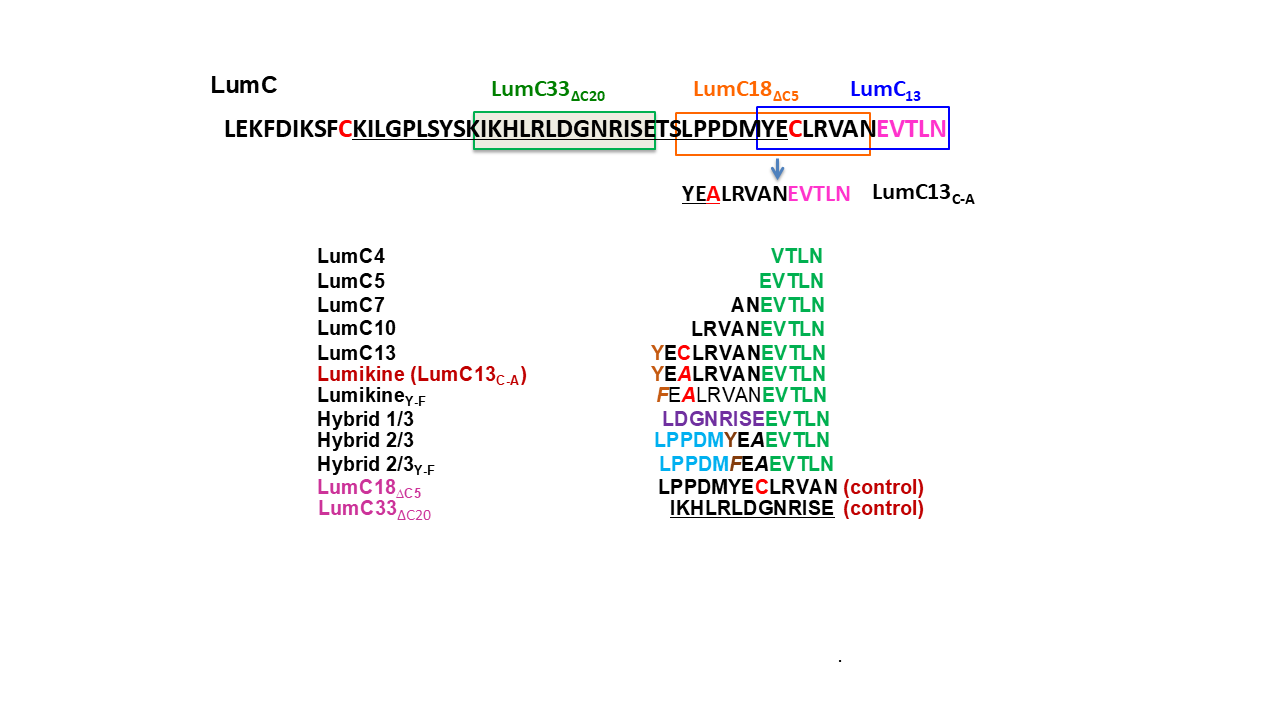

Supplement: Supplementary file 1 [file cells-13-01599-s001.zip › cells-3041284-Suplemenmtary Fig. S1 Sequence of Lumican Peptides.tif]
